# Relationship between the A(8002)G intronic polymorphism of pre-pro-endothelin-1 gene and the endothelin-1 concentration among Tunisian coronary patients

**DOI:** 10.1186/s12872-015-0142-x

**Published:** 2015-11-16

**Authors:** Abdelkader Chalghoum, Yosri Noichri, Azza Dandana, Sana Azaiez, Bruno Baudin, Gouider Jeridi, Abdelhédi Miled, Salima Ferchichi

**Affiliations:** Laboratory of Biochemistry, Farhat HACHED Hospital, Street Doctor Moreau, 4000 Sousse, Tunisia; Valorization and Research Support Space, Center of Biotechnology, Borj Cedria, 2050 Hammam Lif, Tunisia; Department of Biochemistry, Saint-Antoine Hospital, 184 Faubourg Saint-Antoine, 75571 Paris Cedex 12, France; Department of Cardiology, Farhat HACHED Hospital, Street Doctor Moreau, 4000 Sousse, Tunisia

**Keywords:** Acute coronary syndrome, Endothelin-1, Risk factors, Genetic polymorphism

## Abstract

**Background:**

Acute coronary syndromes (ACS) are complex and polygenic diseases which are a real problem of public health. These syndromes require multidisciplinary studies to understand the pathogenesis mechanisms. Our study aims to evaluate the endothelin-1 (ET-1) serum concentration in Tunisian coronary compared to controls healthy, as well as the study of the impact of an intronic polymorphism A (8002) G of pre-pro-endothelin-1 Gene (inactive precursor of ET-1) on the change in serum endothelin-1 and in the susceptibility to Acute coronary syndrome (SCA).

**Methods:**

Our samples were subdivided into coronary patients (157) and healthy subjects (142). The quantification of the ET-1 concentration was performed by high performance liquid chromatography, the identification of the different genotypes of the polymorphism A(8002)G was made by PCR-RFLP. The association between the ET-1 concentration and identified genotypes was realized by adapted software for descriptive statistics, Statistical Package for the Sociological Sciences (SPSS v 21.0).

**Results:**

Our study showed that the concentration of ET-1 was significantly higher in patients compared to controls and that the mutated allele prevails in patients F (G) = 0.78 and there is a minority in controls F (G) = 0.3. Secondly the homozygous genotype GG is associated with higher concentrations of ET-1 in patients and controls, heterozygous genotype AG is associated with intermediaries’ values and AA genotype is related to lower values.

**Conclusion:**

Although the polymorphism studied is an intronic polymorphism, it is involved in the change in serum concentration of ET-1 and is a candidate gene in susceptibility to SCA. Cardiovascular diseases are “polygenic” pathology and do not obey of the law for transmission of Mendel.

## Background

Endothelin-1 (ET-1) is an endothelium derived, potent vasoconstrictor peptide of 21 amino acids (2.5 kDa with a great structural homology with snake venom, the S6 sarafotoxin). It was isolated and sequenced from endothelial cell culture by Yanagisawa in 1988. Its peptide is strongly implicated in the coronary artery diseases genesis and complications by its vasoconstrictors effects [1–3].

This peptide is synthesized as a precursor inactive for 212 amino acids (pre-pro-endothelin-1) then undergoes an enzymatic activation cascade to give mature endothelin-1 of 21 amino acids [3–5].

At the molecular level, Pre-pro-endothelin-1 is expressed by a gene with 9 kb located in chromosome 6 (6p24.1) and composed of 5 exons and 4 introns [6, 7].

In this context, the aim of our study was to quantify the serum concentrations of ET-1 among Tunisian coronary patients compared to healthy controls, and study the impact of an intronic polymorphism A (8002) G (intron 4) of the pre-pro-endothéline-1 gene in the serum concentration of ET-1 and the susceptibility to coronary syndromes.

## Methods

### Populations study

This is a retrospective case-control study, in which, sampling was carried between January 2010 and November 2011. One hundred fifty seven Tunisian coronary subjects (121 men and 36 women) middle-aged (64.8 ± 11.7 years), chosen according to the clinical data, essentially electrocardiogram. One hundred forty two healthy subjects (111 men and 31 women) middle-aged (56.8 ± 9.4 years), without heart disease or other and they are a lot less exposed to cardiovascular risk factors and are not under any treatment, were considered as the healthy group.

The patients and healthy subjects signed a free and clear consent which explain the objectives of this work with an undertaking not to publish the names of participants, their personal data including test results (following the instructions of the Tunisian Medical Ethics Committee, consistent with the Helsinki Declaration).

The approval of compliance with the Helsinki declaration was signed in December 2009 by Mrs the Dean of Faculty of Pharmacy of Monastir, Tunisia, as a member of the Tunisian Medical Ethics Committee.

A datasheet had been prepared for each subject (patient or control) to identify cardiovascular risk factors and know the susceptibility degrees to ACS. This sheet contains the anthropometric characteristics, the biological parameters, the risk factors, the treatments of patients, inclusion criteria (unstable angina and myocardial infarction), exclusion criteria (cancer, thyroid diseases).

### Laboratory analysis

A collection of three tubes were made for each patient and control: one tube without anticoagulant for ET-1 concentration, lipid profile and apolipoprotein the second one is EDTA tube for genomic analysis and the third with Heparin Lithium for glycemia.

A simple biochemical investigation (blood glucose, lipid profile) was done by colorimetric essay (Randox-Antrim, UK) and apolipoprotein measurements by an immunonephelemetry essay (Cobas Integra 400, Roche) to explore the risk of exposure to acute Coronary syndrome in patients and witnesses.

After plasmatic ET-1 extraction with ethanol, the concentration was measured by High Performance Chromatography, coupled to Mass Spectrometry (3200 Q TRAP LC/MS/MS system), according to the Walczak M protocol, 2010 [8], using a synthetic standard (ET-1 Sigma-Aldrich St. Louis, MO, USA) for the specific spectra identification and for the calibration curve determining. Chromatographic separation was carried out with C18 analytical column (30 mm × 2.1 mm, 3.5 μm, Waters Ireland) set at 20 °C. Two solvents mixtures were used: solvent A: Acetonitrile and solvent B: H_2_O. The following gradient was used: 0-5 min 0-100 % A; 5–7 min 100 % A; 7–8, 100–0 % A; 8–15 min, 100 % B. The flow rate was set at 300 μL.min^−1^ and a sample volume of 25 μl was injected in the analytical column.

Genomic DNA was isolated from peripheral leukocytes by a standard technique using Phenol-Chloroform and Proteinase K. The G(8002)A polymorphism in intron 4 in pre-pro-endothelin-1 gene was detected according to our protocol RFLP-PCR product (primers 5’-CAA ACC GAT GTC CTC TGT A-3’ and 5’-ACC AAA CAC ATT TCC CTA TT-3’). The PCR reaction was performed in a total volume of 25 μL containing 3 μl 10 × PCR buffer, 3 mM MgCl_2_, 0.2 mmol/L dNTPs, 0.25 μmol/L each primer, 150 ng of genomic DNA and 1 U of Taq DNA polymerase (Promega). The PCR conditions were 95 °C for 90 s, followed by 35 cycles of 30 s at 95 °C, 45 s at 56 °C and 30 s at 72 °C, with a final elongation at 72 °C for 7 min. The PCR product was digested by restriction analysis with Taq I (T↓CGA) on electrophoresis gel with ethidium bromide. Three genotypes GG, 150 and 208 bp, GA, 358, 150, and 208 bp, and AA, 358 bp were identified.

### Statistical analysis

Database management and statistical analyses were carried out using SPSS (Statistical Package for the Sociological Sciences), version 21.0. Results are presented as means ± SD, or percentages. Means were compared using Student test. The significance threshold was set at 5 % (p < 0.05). The impact of the mutated alleles and genotypes was identified by a Chui square test.

Our study escapes from Hardy-Weinberg model for two major reasons:

The first is that our sampling is a selective sampling (patients with genetic risk factor and healthy controls unexposed to genetic cardiovascular factors). The second is the dominance of the consanguinity (union between first cousins) in the Tunisian population.

## Results

Clinical characteristics and biochemical parameters of patients with coronary artery syndrome and control groups are illustrated in Table 1. No significant difference was observed between the 2 groups in the basis of mean of age and sex. Among cardiovascular risk factors, diabetes, hypertension, smoking and dyslipidemia are greatly mentioned in patients compared with healthy subjects. The disorder of apolipoproteins was observed in patients, there was a significant increase in Apo B serum level and a decreased in serum Apo A-1 level compared with serum apolipoprotein levels in healthy subjects.

**Table 1:** Clinical and biochemical features of patients with ACS and controls subjects

	Patients (*n* = 157)	Control subjects (*n* = 142)	*P*
Age (x ± σ years)	64.8 ± 11.7	56.8 ± 9.4	NS
Sex . Men (%)	121 (77 %)	111 (78.2 %)	NS
Women (%)	36 (23 %)	31 (21.8 %)	NS
BMI (kg/m^2^)	27.6 ± 4	23.3 ± 2.2	NS
Hypertension (%)	88	0	-
Obesity (%)	52	8.5 %	-
Diabetes (%)	64	0	-
Smoking (%)	62.4	7	-
Family cardiac history (%)	68	6	-
Personnel cardiac history (%)	66	0	-
Postmenopausal women (%)	100	82	-
Dyslipidemia (%)	40	0	-
Sedentary (%)	43	11	-
Alcohol (%)	33	14	-
*Glucose (x ± σ mmolL/)*	9.8 ± 4.2	5.40 ± 0.84	<0.0001
*TC (x ± σ mmol/L)*	5.70 ± 3.1	4.60 ± 2.6	<0.001
*HDL-C (x ± σ mmol/L)*	1.14 ± 0.22	1.30 ± 0.41	NS
*LDL-C (x ± σ mmol/L)*	3.60 ± 2.16	2.80 ± 1.4	<0.001
*TG (x ± σ mmol/L)*	1.60 ± 0.9	1.24 ± 0.3	NS
*ApoA1 (x ± σ. g/L)*	1.41 ± 0.62	1.80 ± 0.2	<0.00001
*ApoB (x ± σ. g/L)*	1.40 ± 0.81	0.70 ± 0.2	<0.00001
Treatment: ACA-1 inhibitors (%)	97	0	-
Statins (%)	38	0	-
Beta-Blockers (%)	33	0	-
Ca-Blockers (%)	27	0	-
Diuretics (%)	17	0	-

On the other hand, the ET-1 concentration was significantly higher in patients compared to healthy subjects. Table 2 shows the variation of the serum concentration of ET-1 in healthy subjects and in patients according to cardiovascular risk factors. This investigation has shown that this vasoconstrictor depends on gender, arterial hypertension, tobacco, obesity and dyslipidemia, unlike diabetes, alcohol and physical inactivity.

**Table 2:** The variation of the ET-1 concentration among patients according risk factors

Populations and risk factors	ET-1 (nmol/L)		*P*
Population	Patients (*n* = 157)	15.2 ± 5.3	<0.00001
	Controls (*n* = 142)	7.1 ± 2.7	
Gender	Men (n = 121)	17.4 ± 4.6	<0.00001
	Women (*n* = 36)	7.8 ± 2.3	
Hypertension	Yes (*n* = 138)	16.4 ± 3.3	<0.00001
	No (*n* = 19)	6.6 ± 1.9	
Diabetes	Yes (*n* = 101)	15 ± 5.1	NS
	No (*n* = 56)	15.6 ± 3.8	
Tobacco	Yes (*n* = 98)	11.6 ± 3	<0.00001
	No (*n* = 59)	21.2 ± 6.1	
Obesity	Yes (*n* = 82)	12.2 ± 5.3	<0.001
	No (*n* = 75)	18.2 ± 4	
Dyslipidemia	Yes (*n* = 63)	11.8 ± 1.6	<0.00001
	No (*n* = 94)	17 .5 ± 4.6	
Alcohol	Yes (*n* = 52)	16 ± 4.1	NS
	No (*n* = 105)	14.8 ± 3.8	
Sedentarity	Yes (*n* = 67)	15.7 ± 3.7	NS
	No (*n* = 90)	14.9 ± 2.4	

**Fig. 1:**
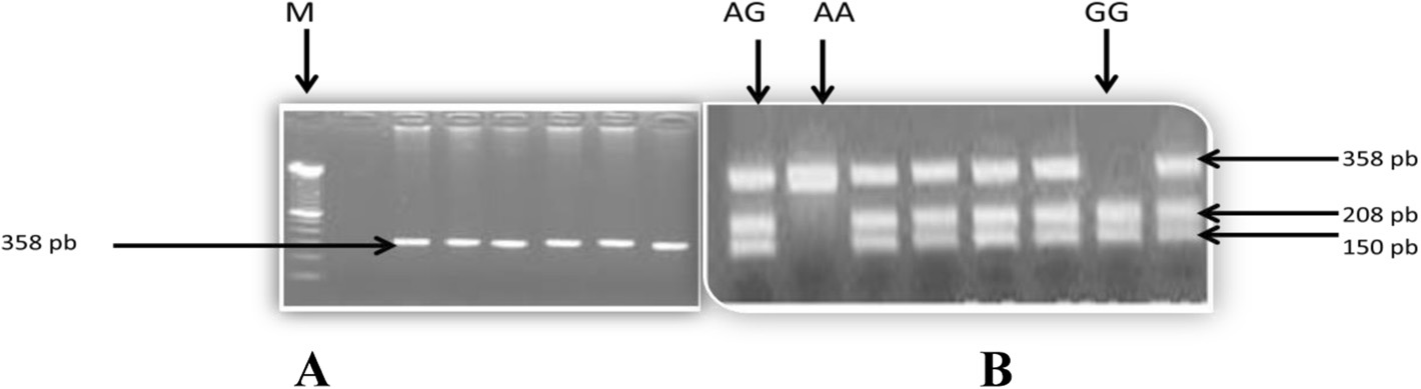
Genotypes identification by RFLP-PCR (on 2 % agarose gel): **a** before enzymatic digestion, single band, with 358 bp corresponding to “intact intron 4”, **b** post enzymatic digestion of PCR products, with the different genotypes (AA with 358 bp, AG with 358 bp + 208 bp + 150 bp and GG with 208 bp and 150 bp). M: molecular weight ladder (100 bp); pb: base pairs; PFLP-PCR: restriction fragment length polymorphism-Polymerase Chain Reaction

Our molecular study shows that the mutated allele G prevails in patients with a frequency F (G) = 0.78 and the non-mutated allele A is majority in control group with a frequency F (A) = 0.65 (Table 3). The identification of genotypes (by RFLP- PCR) is illustrated in Fig. 1.

**Table 3:** Genotypes (associated with the ET-1 serum level) distribution among patients and controls

	Allele frequencies	Genotypes	ET-1 (nmol/L)	*P*
Patients (157)	F(A) = 0.22	AA (12 %)	10.38 ± 4.6	a NS
	F(G) = 0.78	AG (20 %)	12.33 ± 3.1	b 0.001
		GG (68 %)	16.9 ± 4.2	c 0.001
Controls (142)	F(A) = 0.65	AA (54 %)	5.61 ± 1.3	a 0.001
	F(G) = 0.35	AG (22.5 %)	7.27 ± 1.2	b 0.001
		GG (23.5 %)	9.3 ± 2.8	c 0.001

Independence Chi-square test proves that the GG genotype is associated with ACS, *χ*^2^ = 58.75 > > 3.841 (degree of liberty = 1 and α =3.841).

The same test proves the implication of G allele in the disease, *χ*^2^ = 60.69 > > 5.999 (degree of liberty = 2 and α = 5.999).

**Fig. 2:**
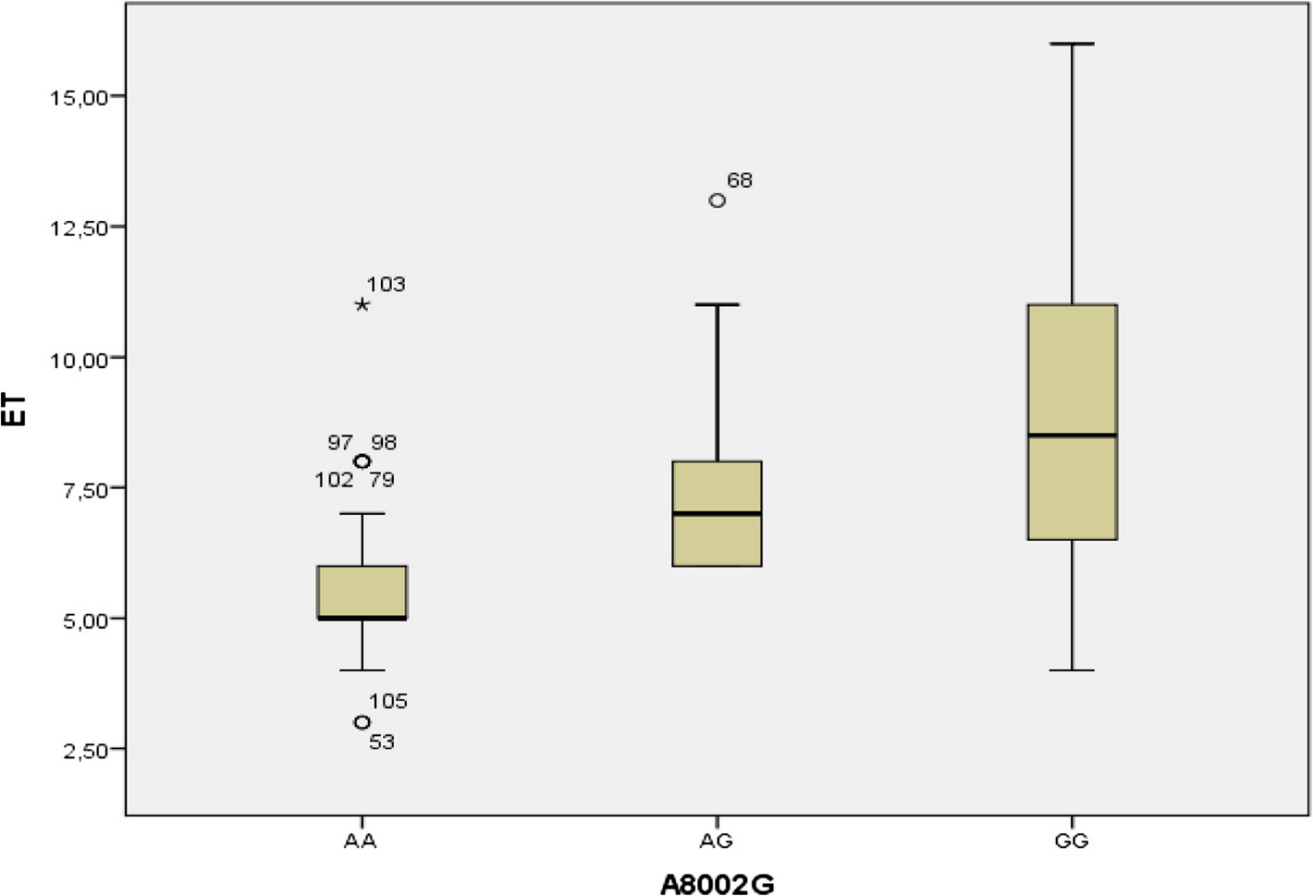
The serum concentration of ET-1 according to different genotypes among control group

**Fig. 3:**
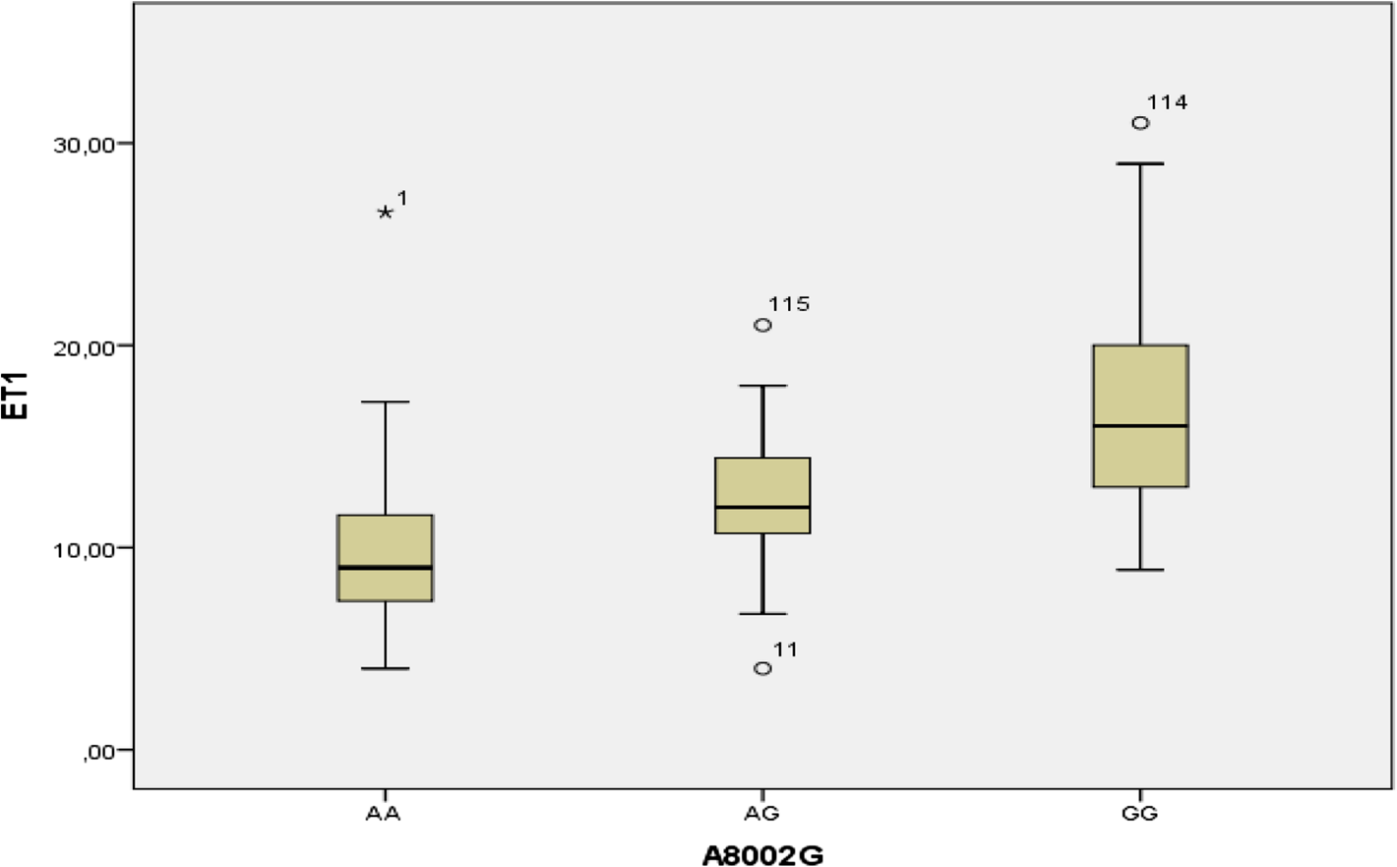
The serum concentration of ET-1 according to different genotypes among patients group

The different genotypes distribution between patients and healthy group, and the association between genotypes and serum level of ET-1 are shown in Table 3, Figs. 2 and 3 respectively.

This association showed that the mutated homozygous genotype is associated with higher serum values of ET-1, that the non-mutated homozygous genotype is associated with lowest values and heterozygous, while AG genotype is linked to intermediate values, this observation is valid for patients and healthy group.

## Discussion

Varied risk factors among patients confirm the multifactorial and polygenic origin of the ACS, well explained by the Framingham study. The lipid profile of patients was partially balanced because the required diet and hypolipidemic treatment, essentially the angiotensin-1 converting enzyme inhibitors with its beneficial effects against metabolic syndrome and lipid complications [9, 10].

The increased concentrations of apolipoprotein B (although 38 % of patients are under statins) consolidate its atherogenic effects and its involvement in the ACS genesis, whereas Apo A1 (is statistically higher in controls) has a cardio-protective effect supported by various studies [11–13].

Our study shows a statistically significant increase in the ET-1 values among patients compared to control subjects (although 33 % of patients are under Beta-Blockers and 27 % are under Ca-Blockers generally used, in this advanced stage of coronary syndrome for reduce the mortality of coronary, particular which have a myocardial infarction), which reflects its role not only in a prolonged vasoconstriction but also in its atherogenic impact, its pro-oxidant effect (indirectly involved in the NADPH oxidase activation and radical oxygen species generation) and its pro-aggregating effect (by its role in the thromboxane A2 synthesis) [5, 14, 15].

The ET-1 level depends, in this study on gender, hypertension, smoking, obesity and dyslipidemia, unlike diabetes, alcohol and physical inactivity.

The high concentration of ET-1 among men patients compared to women patients shows the effect of the male gender as an un-modifiable cardiovascular risk factor.

This increase of ET-1 level has a metabolic origin and non-genetic, since our peptide is expressed by an autosomal gene and the same actors and cleavage and maturation enzymes (endothelin-1 converting enzyme…) are also derived from autosomal genes [8, 16, 17].

The elevated concentration of ET-1 among hypertensive patients compared with normotensive patients is explained by the vasoconstrictor effect of ET-1 and its roles in the sodium reabsorption, in hyperactivity these regulatory effects become hypertensive [17–19].

The ET-1 concentration was inhibited by smoking, obesity and dyslipidemia, this inhibition is explained in the literature by the roles of these factors in the genesis and complications of metabolic syndrome. This latter, generates a liver impuissance in degradation and neutralization of cytotoxic elements that infect the endothelium, main secretory tissue of ET-1.

Otherwise, nicotine has a harmful effect on the endothelium and prevents secretion of endothelial factors [20–22].

The dominance of the mutated allele G in patients proves the effect of this allele as a genetic component to susceptibility to acute coronary syndrome. This dominance has resulted in a majority of patients with homozygous genotype GG (highly exposed to risk) and heterozygous AG patients intermediately exposed (single dose exposure from for patients with AG and double dose exposure for patients with GG).

For the minority of patients non-carriers of the mutated allele (12 %), we can confirm that the provenance of disease is linked to other factors (which again proves the diverse origin and polygenic nature of ACS).

For healthy group, the majority carries the non-mutated genotype AA, the presence of healthy subjects AG or GG proves the non Mendelian nature of this polymorphism.

The G allele among controls can be called “silent risk marker or candidate allele currently» neutral” [23–26].

The association between G allele and coronary risk is explained by the association between the G allele and high level of ET-1 in patients and healthy subjects (Figs. 2 and 3). The GG genotype is associated with elevated values of ET-1 in patients and controls, while the AG genotype is associated with intermediate values in both populations and AA genotype is associated with the lowest values. Although this polymorphism is an intronic polymorphism (it does not affect the final structure of the protein) its functionality is caused by the role of introns in the regulation of the protein expression level (they fix the transcription factors), an intronic aberration may amplify or inhibit the level of protein expression [5, 27].

## Conclusion

ACS is a complex disease that does not obey of the Mendel transmission law, the pathophysiology is caused by an overlap between genetic and behavioral factors (smoking, physical inactivity …). A good understanding of coronary pathophysiology requires multidisciplinary studies of risk factors and candidate genes, their degree of involvement and linkage disequilibrium between them.

## Abbreviations

*ACS*: acute coronary syndrome
*Apo A*: apolipoprotein A1
*Apo B*: apolipoprotein B
*ET-1*: endothelin-1
*RLFP-PCR*: restriction fragment length polymorphism-polymerase chain reaction
*SPSS*: Statistical Package for the Sociological Sciences
